# The Treatment of the Umbilical Cord

**Published:** 1898-10

**Authors:** 


### THE TREATMENT OF THE UMBILICAL CORD.

Paul Bar (*Journal des Praticien*) asks these questions:

1. Should the cord be tied? 2. When should it be tied? 3 How should it be tied? and answers them as follows:

1. The cord should be tied because, although the probabilities of umbilical hemorrhage are very small, a certain proportion of mischances exist, and these should be guarded against. 2. In some cases not a moment's delay is admissible, as, for instance, when the head is born and the shoulders are engaging the perinuem, while one or more turns of the cord are around the neck. The cord should then be clamped by two forceps and divided between them. It can be cut and tied in the proper place at leisure. Or, a child is born apparently dead. The cord should then be divided immediately so as to afford opportunity for paying the necessary attention to attempts at restoration. But if everything has been normal a delay of five or six minutes is recommended. If the cord is immediately cut, there will be seen oozing from the placental extremity a certain quantity of blood, which would have passed into the child. If this blood be allowed to drip upon the pan of a balance, the pan will be found to sink for five minutes or so from the weight of this lost blood of which the child has been robbed, and this amount averages from about nine hundred to thirteen hundred and fifty grains. 3. The author's method of compressing the cord is by means of forcible pressure forceps, sterilized by heat. The forceps is then surrounded by a pad of absorbent cotton and left for from thirty-six to forty-eight hours underneath the binder.—*N. Y. Med. Jour.*

